# Cellars

**Published:** 1859-02

**Authors:** 


### CELLARS.

THERE ought to be no cellar in any family dwelling. The house should be one or two feet above ground, with a trench around it a foot deep, so that the surface of the earth immediately under the floor should be always kept dry to the depth of several inches, and there should be open spaces in the

"under-pinning," so as to allow a free circulation of air at all times.

New York has the reputation of being about the sickliest city in the world—that is, a larger number of persons die in it during a year, in proportion to the population, than in any other first-class city in Christendom, the mortality of which is reliably reported.

There is reason to believe that moral causes originate a very large number of the deaths in New York city every year. But among the physical causes is the faulty construction of dwelling-houses, and no unimportant item is the cellar, which is under the whole building, its floor being, on an average fourteen feet beneath the level of the street. The only door of the cellar opens into the lower hall or passage. Through this door the servants pass many times every day for fuel and the ordinary articles of cooking, and at every opening a strong current of air rushes and passes upward, and impregnates every room of the building. That air is always close, raw and damp, and saturated with the effluvia given out by decaying vegetables, bones, meats, rotten wood, and offall of every conceivable description; for be it remembered that the larger houses are so contrived that, by a convenient arrangement, the ashes from the kitchen fire, with all the articles swept from a a kitchen floor, or usually thrown into a kitchen fire-place, are let down into the cellar into one promiscuous heap, to be cleaned out in the spring, or fall, or both. We have seen half a dozen cart loads borne away at a single time from five-story brown stone fronts. In addition, many houses are so constructed, that all the water from the kitchen, dish-water, wash-water, soap suds, floor washings, and the like, pass into the "sink," as it is called, which is in the cellar, which is a hole dug in the earth or sand, and covered over, to be passed off into the street drain; but, before it passes off, the earth becomes saturated, and a noisome effluvia is always rising day and night, winter and summer.

Still further, our magnificent mansions have the privy under one and the same roof with cellar, chamber, and parlor; and that its sink should not become saturated, and that its effluvia should not arise more or less, or in some other manner make its way into the cellar, is an impossibility.

That such arrangements should prevail in three houses out of four in an intelligent community is certainly not very creditable.

Not long ago we had occasion to go into the cellar of a store on Broadway, near the Park, and, in looking for some article, we had occasion to pass the privy of the establishment, which was immediately under the grating over which every person had to pass to enter the store. The sights on wall, floor, seats, &c., were simply incredible; yet into this temple of filth gentlemanly proprietors and well-dressed clerks enter often daily, and within the next three minutes are chatting at the breadth of half a counter with the fashion of New York!

In houses already built, we suggest that a hole six, eight, or ten inches square, be cut in or near the cellar ceiling, leading at some distance up into the chimney, where, meeting with the hot air, a forcible draft would be made upwards and outwards, and thus secure a constant and thorough cellar ventilation. Every family should, in addition, fasten up the internal cellar entrance, and let it be from without the house through a door opening into the yard or back area, and thus make it impossible for the foul air of the cellar to find its way into the sitting rooms and chambers of the whole household.

